# Supplementary material for: CRIF1 siRNA-Encapsulated PLGA Nanoparticles Suppress Tumor Growth in MCF-7 Human Breast Cancer Cells
Source: Int J Mol Sci. 2023 Apr 18;24(8):7453. doi: 10.3390/ijms24087453 (PMC10138627; doi:10.3390/ijms24087453)
Supplement: Supplementary file 1 [file ijms-24-07453-s001.zip › ijms-2328561-supplementary.pdf]

Supplementary Figure 1

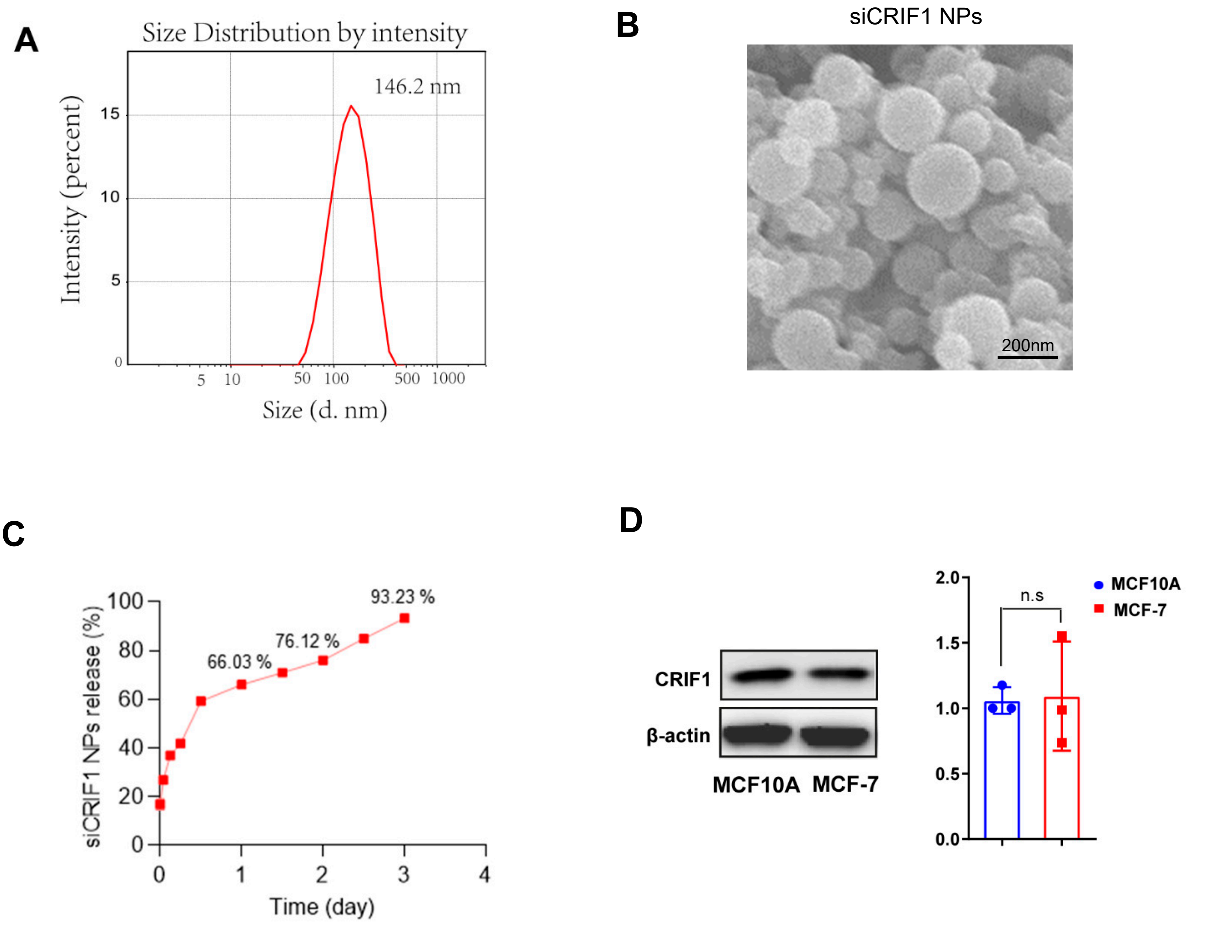

Supplementary Figure S1.
(A) Characterization of the size of CRIF1 siRNA-encapsulated PLGA nanoparticles. (B) CRIF1 NPs were investigated by scanning electron microscopy. Scale bar = 200 nm. (C) Amount of siCRIF1 NPs release into PBS. (D) CRIF1 protein expression was measured by immunoblotting. The data are presented as means ± SEM of three independent experiments.
